# New use of low-dose aspirin and risk of colorectal cancer by stage at diagnosis: a nested case–control study in UK general practice

**DOI:** 10.1186/s12885-017-3594-9

**Published:** 2017-09-07

**Authors:** Luis A. García Rodríguez, Montse Soriano-Gabarró, Susan Bromley, Angel Lanas, Lucía Cea Soriano

**Affiliations:** 1 Spanish Centre for Pharmacoepidemiologic Research, c/ Almirante 28, 2°, 28004 Madrid, Spain; 2 Epidemiology, Bayer AG, Müllerstr. 178, 13353 Berlin, Germany; 3 EpiMed Communications Ltd, Abingdon, Oxford, OX14 1QS UK; 4 London School of Hygiene and Tropical Medicine, Keppel St, London, WC1E 7HT UK; 5 Servicio de Aparato Digestivo, Hospital Clínico, University of Zaragoza, IIS Aragón, Zaragoza, Spain; 6 CIBERehd, Av. Monforte de Lemos, 3-5. Pabellón 11. Planta 0, 28029 Madrid, Spain; 7 Department of Preventive Medicine and Public Health, Faculty of Medicine, Complutense University of Madrid, Av. Séneca, 2, 28040 Madrid, Spain

**Keywords:** Colorectal cancer, Aspirin, Nested case-control studies, Chemoprevention, Diagnosis

## Abstract

**Background:**

Evidence from clinical trial populations suggests low-dose aspirin reduces the risk of colorectal cancer (CRC). Part of this reduction in risk might be due to protection against metastatic disease.

**Methods:**

We investigated the risk of CRC among new-users of low-dose aspirin (75–300 mg), including risk by stage at diagnosis. Using The Health Improvement Network, we conducted a cohort study with nested case–control analysis. Two cohorts (*N* = 170,336 each) aged 40–89 years from 2000 to 2009 and free of cancer were identified: i) new-users of low-dose aspirin, ii) non-users of low-dose aspirin, at start of follow-up, matched by age, sex and previous primary care practitioner visits. Patients were followed for up to 12 years to identify incident CRC. 10,000 frequency-matched controls were selected by incidence density sampling where the odds ratio is an unbiased estimator of the incidence rate ratio (RR). RRs with 95% confidence intervals were calculated. Low-dose aspirin use was classified ‘as-treated’ independent from baseline exposure status to account for changes in exposure during follow-up.

**Results:**

Current users of low-dose aspirin (use on the index date or in the previous 90 days) had a significantly reduced risk of CRC, RR 0.66 (95% CI 0.60–0.74). The reduction in risk was apparent across all age groups, and was unrelated to dose, indication, gender, CRC location or case-fatality status. Reduced risks occurred throughout treatment duration and with all low-dose aspirin doses. RRs by aspirin indication were 0.71 (0·63–0·79) and 0.60 (0.53–0.68) for primary and secondary cardiovascular protection, respectively. Among cases with staging information (*n* = 1421), RRs for current use of low-dose aspirin were 0.94 (0.66–1.33) for Dukes Stage A CRC, 0.54 (0.42–0.68) for Dukes B, 0.71 (0.56–0.91) for Dukes C, and 0.60 (0.48–0.74) for Dukes D. After 5 years’ therapy, the RR for Dukes Stage A CRC was 0.53 (0.24–1.19).

**Conclusions:**

Patients starting low-dose aspirin therapy have a reduced risk of Stages B–D CRC, suggesting a role for low-dose aspirin in the progression of established CRC; a substantial reduction in the risk of Dukes A CRC may occur after 5 years’ therapy.

**Electronic supplementary material:**

The online version of this article (10.1186/s12885-017-3594-9) contains supplementary material, which is available to authorized users.

## Background

Colorectal cancer (CRC) is the third most commonly diagnosed cancer worldwide, with more than 1.3 million new cases reported in 2012 [1]. Long-term (up to 20 years) follow-up of cardiovascular trials have demonstrated, in post hoc analyses, that patients randomized to daily low-dose aspirin (75–300 mg) have a reduced risk of CRC incidence and mortality after a delay of several years [2]. The hypothesis that aspirin has a chemopreventive effect early in the adenoma sequence in CRC development is consistent with this latency period, and is supported by findings from randomized controlled trials (RCTs) showing daily low-dose aspirin (81–325 mg) reduces colorectal adenoma recurrence in average/high-risk populations [3].

In early cardiovascular trials, the protective effect of low-dose aspirin against CRC was greatest in patients with longer scheduled duration of trial treatment [2]. However, analyses were limited based on unknown aspirin exposure during the post-trial follow-up period, and inability to adjust for confounders. Patients who discontinued aspirin in the intervention arm or started treatment in the placebo arm after the randomized phase would have been analyzed according to exposure status at randomization, thus the protective effect of aspirin could have been mainly due to chronic aspirin exposure over the relatively short in-trial period. Recent analyses of in-trial data show that aspirin substantially reduces metastatic CRC at initial diagnosis [4], which cannot be accounted for by an effect early in the adenoma–carcinoma sequence. Observational data suggest aspirin may reduce the risk of metastatic breast [5] and prostate [6] cancer at initial presentation, and reduced mortality has been reported for CRC [7] and breast cancer [8] with aspirin use following diagnosis. Aspirin may therefore have an inhibitory effect on the growth and spread of tumours as well as on their initial development, and this effect could be explored further in real-world patients by the evaluation of low-dose aspirin on the risk of different stage CRC in clinical practice.

## Methods

Using a UK primary care database of electronic medical records (EMRs), we carried out a cohort study with nested case–control analysis to evaluate the association between use of low-dose aspirin and risk of incident CRC, both overall and by aspirin dose, duration of use and indication. We also aimed to assess the overall association between low-dose aspirin and CRC risk by CRC stage at diagnosis, site and case-fatality status, as well as among patients with or without previous bowel investigations (colonoscopy/sigmoidoscopy). We hypothesized that the strength of association between low-dose aspirin and CRC would vary by Dukes stage at diagnosis. An independent scientific review committee for THIN (reference number 12-044 V) approved the study protocol.

### Data source

We used The Health Improvement Network (THIN) database, which contains computerized primary care data for ~6% of the UK population [9] and is demographically representative of the UK population as a whole [10]. Clinical data are entered as part of routine patient care using Read codes [11], although further details can be added as free text, and prescriptions are automatically recorded upon issue. Further details about the database are described elsewhere. [12, 13] At the time the study was carried out, approximately one fifth of practices contributing to THIN were linked to Hospital Episode Statistics (HES) [14], thus information from secondary care could be obtained for individuals in these practices.

### Source population and identification of the two study cohorts

The source population included individuals in THIN aged between 40 and 84 years from 1 January 2000 to 31 December 2009 who met the following data quality and completeness standards: at least 2 years of registration with the general practice, a minimum of 1 year since the beginning of computerized prescription history, and a minimum of one health encounter in the previous 3 years. Individuals with a prescription for low-dose aspirin or a diagnosis of cancer any time before the study entry were excluded. From within the source population we identified all new-users of low-dose aspirin (*N* = 170,336) and matched each one to an individual still free of low-dose aspirin on that day (1:1 matching). Matching criteria were age, sex and PCP visits in the previous year (as a proxy for general health/comorbidity and as an attempt to control for differences between users and non-users of aspirin at the start of follow-up that are otherwise difficult to control). Details on the identification of the two cohorts can be found in Additional file 1). The matching date was set as the start date (start of follow-up) for the identification of incident CRC cases.

### CRC case identification and validation

**Fig. 1:**
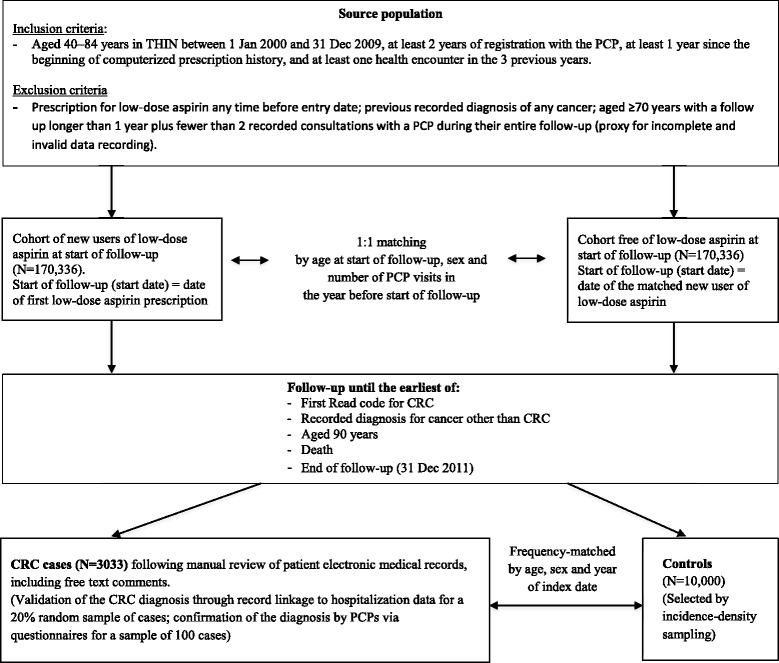
Flowchart depicting the nested case–control study design. CRC, colorectal cancer; PCP, primary care practitioner

Both study cohorts were followed-up from the start date until a Read code suggestive of CRC (see Additional file 2), a recorded diagnosis of another cancer, age 90 years, death, or end of the study period (31 December 2011; Fig. 1), whichever came first. The maximum duration of follow-up was therefore 12 years. A total of 3805 individuals had a CRC Read code during follow-up, and had their EMRs (with free text comments) manually reviewed while masked to aspirin exposure [13]. Patients were deemed incident cases unless there was evidence from the patient record suggesting otherwise; for example, a prevalent case, an uncertain diagnosis following biopsy results, or presence of a second primary cancer either concurrently or previously. Information relating to the CRC diagnosis was extracted, including (where available) details on CRC type (colon or rectum), stage (available in the free-text comments only), surgery related to the event, adjuvant therapy treatment, and procedures involved in the diagnosis (e.g. colonoscopy, sigmoidoscopy). The index date was the earliest of the following: the date of the first CRC-related symptom, the date of screening/diagnostic procedure, or the date of surgery. In cases where the index date was unclear (for instance, when patients presented several times to their PCP with non-specific symptoms), an external gastroenterologist was consulted to ascertain the most likely index date. In the majority of CRC cases (83%), the index date was backdated from the date of the recorded CRC diagnosis; the mean number of backdated days was 56.6 and the median was 36.0. Two external data sources were used to validate the incident CRC cases ascertained in THIN following the manual review process: 1) questionnaires sent to PCPs (for a random sample of 100 cases), and 2) HES data for individuals in linked practices. Findings from the validation process have been published previously [13].

### Control selection

Ten thousand controls were randomly sampled and frequency-matched to CRC cases by age, sex, and year of the index date. Incidence density sampling was used where the likelihood of being selected as a control was proportional to the individual’s person-time at risk. All eligibility criteria applied to cases were also applied to controls.

### Statistical analysis

Nested case–control analysis was carried out using cases and controls arising from the two cohorts to evaluate the association between low-dose aspirin use and other potential risk factors (see Additional file 1 for further details), and CRC. Odds ratios (ORs) with 95% confidence intervals (CIs) were calculated using unconditional logistic regression. By using incidence density sampling, the OR is an unbiased estimator of the incidence rate ratio (RR) [15]. Drug treatment was categorised as follows: *current use*, use on the index date or in the previous 90 days; *recent/past use*, use ≥91 days before the index date; and *non-use*, when there was no prescription any time prior to the index date. Using these definitions of actual aspirin use in relation to the index date, rather than aspirin exposure determined solely at the start of follow-up, enabled us to account for changes in individuals’ low-dose aspirin exposure over time, which could happen with a long follow-up period such as in this study. Any member of either study cohort could have used low-dose aspirin at or before the index date, not just those designated to the low-dose aspirin cohort at the start of follow-up. For example, a member of the non-user cohort may have initiated use of the drug during follow-up and vice versa). Although low-dose aspirin can be obtained over-the-counter (OTC) in the UK, access to healthcare is straightforward and prescriptions are free for individuals aged ≥60 years, which is the age range of the patient population that most commonly uses low-dose aspirin – a factor likely to encourage prescription. Furthermore, our previous validation study in THIN showed the impact of potential misclassification of low-dose aspirin in the database owing to unrecorded OTC aspirin use would be minimal [16]. In addition, as part of another study using the Clinical Practice Research Datalink (CPRD; a similar database to THIN), nearly all chronic low-dose aspirin use was via prescription [17]. Duration of therapy was calculated for current low-dose aspirin users by totalling the individual lengths of all consecutive prescriptions with treatment gaps greater than 90 days considered genuine breaks in therapy. Stratified and sub-group analyses were performed and interaction terms were used to test for potential effect modification by age, sex and CRC stage at diagnosis. Sensitivity analyses were carried out restricting to CRC cases with known CRC stage. Analyses were undertaken with STATA version 12.0.

## Results

### Descriptive findings

Characteristics of both study cohorts at the start of follow-up are presented in Table 1. New users of low-dose aspirin at the start of follow-up had a higher frequency of comorbidities, smokers, and overweight individuals. Similar proportions of patients between the two cohorts had a previous record of a colonoscopy, sigmoidoscopy or a gastrointestinal polyp. Over a mean follow-up of 5.32 years (95% CI: 5.31–5.33), 3303 incident cases of CRC were identified after manual review of patient’s EMRs. Clinical features of the malignancies are shown in Additional file 3 **.** Among those with recorded staging (*N* = 1421), about one third (34.9%) were Dukes Stage D, and a quarter (26.0%) were Dukes Stage C. The mean age of cases was 68 years and 59% were male. Time to CRC occurrence was similar across Dukes Stages (see Additional file 4 **)**. Approximately 80% (*n* = 2433) of CRC cases were still alive at 1-year post index date (non-fatal cases), of which 48% were male. Overall, 54% of cases were still alive at 5 years; 87% of Dukes Stage A cases, and 78%, 57%, 9% and 57% of Dukes Stages B, C, D and unknown stage cases, respectively. Cases with or without recorded staging were similar in terms of demographics, recorded symptoms and diagnostic investigations (see Additional files 5 and 6). Greater use of health care contacts (hospitalizations, referrals and PCP visits) in the year prior to the index date, former smoking and higher alcohol consumption were significant predictors of CRC, while none of the comorbidities evaluated showed a clear association with CRC (Table 2).

**Table 1:** Frequency distribution of comorbidities, lifestyle factors and healthcare use among two study cohorts

Characteristic^b^	Both cohorts *N* = 340,217^a^ n (%)	Non-exposed to low-dose apirin at start of follow-up cohort *N* = 169,992^a^ n (%)	New users of low-dose aspirin at start of follow-up cohort *N* = 170,225^a^ n (%)
Hypertension	80,269 (23.6)	55,228 (32.5)	25,041 (14.7)
Diabetes	46,994 (13.8)	12,365 (7.3)	34,629 (20.3)
COPD	16,677 (4.9)	7891 (4.6)	8786 (5.2)
IBD	4073 (1.2)	2061 (1.2)	2012 (1.2)
Depression	69,241 (20.4)	31,654 (18.6)	37,587 (22.1)
GI conditions	71,961 (21.2)	33,161 (19.5)	38,800 (22.8)
Record of prior colonoscopy, sigmoidoscopy or GI polyp	30,150 (8.9)	14,262 (8.4)	15,888 (9.3)
Smoking			
Non-smoker	150,723 (44.3)	79,726 (46.9)	70,997 (41.7)
Current smoker	67,598 (19.9)	31,157 (18.3)	36,441 (21.4)
Former smoker	103,536 (30.4)	48,552 (28.6)	54,984 (32.3)
Unknown	18,360 (5.4)	10,557 (6.2)	7803 (4.6)
BMI (kg/m^2^)			
15–19	10,581 (3.1)	5976 (3.5)	4605 (2.7)
20–24	86,326 (25.4)	47,714 (28.1)	38,612 (22.7)
25–29	117,293 (34.5)	57,113 (33.6)	60,180 (35.4)
≥ 30	78,335 (23.0)	31,460 (18.5)	46,875 (27.5)
Unknown	47,682 (14.0)	27,729 (16.3)	19,953 (11.7)
PCP visits^c^			
0–1	11,729 (3.4)	5842 (3.4)	5887 (3.4)
2–4	43,481 (12.8)	21,711 (12.8)	21,770 (12.8)
5–9	98,550 (29.0)	49,238 (29.0)	49,312 (29.0)
10–19	130,757 (38.4)	65,353 (38.4)	65,404 (38.4)
≥ 20	55,700 (16.4)	27,848 (16.4)	27,852 (16.4)

*BMI* body mass index, *COPD* chronic obstructive pulmonary disorder, *GI* gastrointestinal, *IBD* inflammatory bowel disease

^a^The number of individuals in each of the two cohorts is slightly lower than the 170,336 originally identified because later information from a more recent version of the database showed that these patients had dropped out of the cohort (information that was not available in earlier versions of the database)

^b^All variables were measured any time before the start of follow-up except for PCP visits, which were collected in the year before the start of follow-up

^c^In the year before the start of follow-up (each new users of low-dose aspirin/non-exposed pair from the two cohorts was matched by number of PCP visits at the start of follow-up, in addition to matching by sex and age at start of follow-up)

**Table 2:** Frequency distribution of comorbidities, lifestyle factors and healthcare use of CRC cases and controls, and their association with risk of CRC

Characteristic	Controls *N* = 10,000 n (%)	Cases *N* = 3033 n (%)	RR (95% CI)^a^
PCP visits^b^			
0–1	486 (4.9)	92 (3.0)	1.00 (−)
2–4	1094 (10.9)	261 (8.6)	1.37 (1.06–1.79)
5–9	2624 (26.2)	699 (23.0)	1.76 (1.38–2.25)
10–19	3715 (37.1)	1164 (38.4)	2.28 (1.79–2.91)
≥ 20	2081 (20.8)	817 (26.9)	3.07 (2.38–3.96)
Referrals^b^			
0–1	4760 (47.6)	1182 (39.0)	1.00 (−)
2–4	2778 (27.8)	917 (30.2)	1.28 (1.16–1.43)
5–9	1716 (17.2)	612 (20.2)	1.35 (1.19–1.53)
≥ 10	746 (7.5)	322 (10.6)	1.57 (1.33–1.85)
Hospitalizations^b^			
None	8496 (85.0)	2424 (79.9)	1.00 (−)
1	930 (9.3)	375 (12.4)	1.32 (1.16–1.51)
2	328 (3.3)	148 (4.9)	1.45 (1.18–1.77)
≥ 3	246 (2.5)	86 (2.8)	1.08 (0.83–1.39)
BMI (kg/m^2^)^c^			
< 20	343 (3.4)	111 (3.7)	1.07 (0.85–1.35)
20–24	2665 (26.7)	774 (25.5)	1.00 (−)
25–29	3793 (37.9)	1144 (37.7)	1.03 (0.93–1.15)
≥ 30	2324 (23.2)	714 (23.5)	1.04 (0.92–1.17)
Unknown	875 (8.8)	290 (9.6)	1.25 (1.06–1.47)
Smoking status			
Non-smoker	4378 (43.8)	1231 (40.6)	1.00 (−)
Current	1292 (12.9)	376 (12.4)	1.07 (0.94–1.23)
Former	4102 (41.0)	1362 (44.9)	1.20 (1.09–1.31)
Unknown	228 (2.3)	64 (2.1)	0.93 (0.69–1.27)
Alcohol consumption (units/week)^d^			
None	1771 (17.7)	483 (15.9)	1.00 (−)
1–9	4722 (47.2)	1401 (46.2)	1.12 (0.99–1.26)
10–20	1521 (15.2)	480 (15.8)	1.21 (1.04–1.41)
≥ 21	725 (7.3)	274 (9.0)	1.46 (1.20–1.78)
Unknown	1261 (12.6)	395 (13.0)	1.15 (0.97–1.36)
Comorbidities^e^			
Diabetes	1852 (18.5)	598 (19.7)	0.98 (0.87–1.10)
IBD	124 (1.2)	40 (1.3)	1.04 (0.72–1.50)
IBS	534 (5.3)	176 (5.8)	1.02 (0.85–1.22)
Gout	765 (7.6)	250 (8.2)	1.10 (0.94–1.29)
Hypertension	5626 (56.3)	1740 (57.4)	1.01 (0.93–1.11)
Hypercholesterolemia	1655 (16.6)	484 (16.0)	0.96 (0.83–1.35)
Upper GI disorders	2165 (21.6)	656 (21.6)	0.93 (0.84–1.03)

*BMI* body mass index, *CI* confidence interval, *CRC* colorectal cancer, *GI* gastrointestinal, *IBD* inflammatory bowel disease, *IBS* irritable bowel syndrome, *NSAIDS* non-steroidal anti-inflammatory drugs, *PCP* primary care practitioner, *RR* rate ratio, *u/w* units per week

^a^Adjusted by the matching variables (age, sex and year of index date) and number of PCP visits, smoking (any time before index date), insulin, NSAIDs, BMI (any time before index date) and oral steroids

^b^In the year before the index date

^**c**^Any time before the index date taking the value recorded nearest to the index date

^d^Units per week = 10 ml (8 g) of pure ethanol

^e^Any time before the index date except for GI disorders, which were ascertained anytime up to and including the start date. Reference group for all comorbidities was not having the respective condition

### Low-dose aspirin use and risk of CRC

**Fig. 2:**
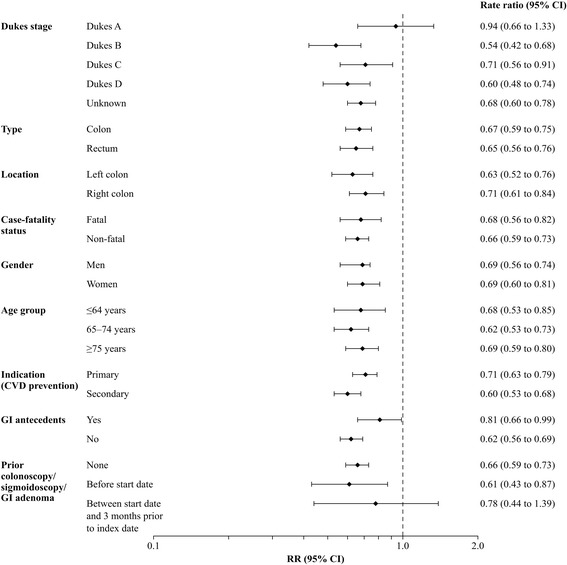
RRs (95% CIs) for the association between current use of low-dose aspirin and risk of CRC by case subgroup and subpopulation. RRs were adjusted for the matching variables (age, sex and year of index date) and number of PCP visits, smoking (any time before index date), insulin, NSAIDs, BMI (any time before index date) and oral steroids.. For all RRs other than those for each Dukes stage, all CRC cases were used irrespective of whether stage was recorded or not recorded (unknown). ^*^Deaths within the first year after CRC diagnosis were considered to be fatal cases. ^†^Record of an adenoma, colonoscopy or sigmoidoscopy. BMI, body mass index; CI, confidence interval; CVD, cardiovascular disease; GI, gastrointestinal, NSAIDS, non-steroidal anti-inflammatory drugs; PCP, primary care practitioner; RR, rate ratio

Associations between use of low-dose aspirin and risk of CRC are presented in Table 3. Current low-dose aspirin users had a 34% statistically significant decreased risk of CRC, RR 0.66 (95% CI: 0.60–0.73). A decreased risk of CRC was observed with all aspirin doses evaluated – 75, 150 or 300 mg/day – although the latter dose was not statistically significant, with no dose–response relationship observed within this range. Among cases and controls, approximately two-thirds of all current low-dose aspirin use was of at least 1-year duration. A reduction in CRC risk was seen throughout treatment duration, with a constant 40% reduction after the first year. Associations between current use of low-dose aspirin and CRC by subpopulation (age, gender, low-dose aspirin indication, prior gastrointestinal antecedents and prior colonoscopy/sigmoidoscopy/gastrointestinal adenoma) as well as by features of the cancer (Dukes’s stage, type [colon or rectum], location and fatality) are shown in Fig. 2). Low-dose aspirin was associated with a 40% statistically significant reduced risk of CRC when used for secondary CVD prevention and approximately a 30% reduced risk when used for primary CVD prevention (Fig. 2). A 30–40% significant protective effect was seen for both fatal and non-fatal cases, for colon or rectal CRC, for both men and women, and across all age groups. Age didn’t modify the protective effect of low-dose aspirin (*p* for interaction = 0.27), and no differences were seen between men and women (*p* for interaction = 0.24). The reduced risk of CRC with low-dose aspirin was also observed among patients with or without GI antecedents, as well as among those with or without previous colonoscopy/sigmoidoscopy/gastrointestinal adenoma.

**Table 3:** Frequency distribution of low-dose aspirin among CRC cases and controls, and RR (95% CI) for their association with risk of CRC

Low-dose aspirin use	Controls *N* = 10,000 n (%)	Cases *N* = 3033 n (%)	RR (95% CI)^a^	RR (95% CI)^b^
Recency				
Non-use	3557 (35.6)	1247 (41.1)	1.00 (−)	1.00 (−)
Current use	4562 (45.6)	1255 (41.4)	0.80 (0.73–0.88)	0.66 (0.60–0.73)
Recent/past use^c^	475 (4.8)	158 (5.2)	0.73 (0.64–0.84)	0.78 (0.64–0.95)
Daily dose^d^				
75 mg	4128 (41.3)	1137 (37.5)	0.78 (0.71–0.86)	0.66 (0.60–0.73)
150 mg	402 (4.0)	107 (3.5)	0.75 (0.60–0.94)	0.62 (0.50–0.78)
300 mg	32 (0.3)	11 (0.4)	0.97 (0.49–1.94)	0.82 (0.41–1.64)
Formulation				
Plain	3716 (37.2)	1008 (33.2)	0.77 (0.70–0.85)	0.65 (0.59–0.72)
Enteric coated	846 (8.5)	247 (8.1)	0.83 (0.71–0.97)	0.70 (0.59–0.82)
Duration of use				
< 1 year	1430 (14.3)	433 (14.3)	0.86 (0.76–0.97)	0.72 (0.63–0.82)
1–5 years	2370 (23.7)	632 (20.8)	0.76 (0.68–0.84)	0.64 (0.57–0.72)
≥ 5 years	762 (7.6)	190 (6.3)	0.70 (0.59–0.84)	0.61 (0.51–0.73)

All estimates are among current users of low-dose aspirin (reference group = non-use) unless otherwise specified

*BMI* body mass index, *CI* confidence interval, *NSAIDS* non-steroidal anti-inflammatory drugs, *PCP* primary care practitioner, *RR* rate ratio

^**a**^Adjusted by the matching factors (age, sex and year of index date)

^**b**^Adjusted by the matching factors (age, sex and year of index date) and number of PCP visits, smoking (any time before index date), insulin, NSAIDs, BMI (any time before index date) and oral steroids

^c^For patients with a duration of use of at least 1 year (25% of all recent/past users)

^d^Refers to the estimated average quantity dose. No appreciable difference in the results were observed when dose of the first prescription during follow-up was used or when the dose of last prescription before the index date was used

Significantly decreased risks of CRC were observed for Dukes Stages B to D; RR 0.54 (95% CI 0.42–0.68) for Dukes Stage B, RR 0.71 (95% CI 0.56–0.91) for Dukes Stage C, and RR 0.60 (95% CI: 0.48–0.74) for Dukes Stage D **(**Fig. 2 **)**, with the reduction in risk seen throughout treatment duration (see Additional file 7). For cases with unknown cancer stage, the RR was 0.68 (95% CI: 0.60–0.78). Colorectal cancer stage at diagnosis was found to significant modify the protective effect of low-dose aspirin (*p* for interaction = 0.03). The RR for Dukes Stage A was 0.94 (95% CI: 0.66–1.33), with no reduction in risk observed with less than 1 year of treatment, but with the suggestion of a substantial risk reduction after 5 years’ therapy, RR 0.53 (95% CI: 0.24–1.19; only seven Dukes Stage A CRC cases had at least 5 years’ low-dose aspirin exposure). Results restricting our analysis to only CRC cases with recorded stage are shown in Additional file 8. In the 1-year lag-time analysis (using all cases irrespective of whether CRC stage was recorded or not recorded; see Additional file 9), current users of low-dose aspirin had an RR of 0.82 (95% CI: 0.74–0.92) with the risk estimate lower with more than 5 years’ treatment duration, RR: 0.72 (95% CI: 0.58–0.89).

## Discussion

Patients starting treatment with low-dose aspirin had a significant 34% decreased risk of developing CRC compared with those not taking low-dose aspirin, in line with previous findings of a chemopreventive effect of aspirin against CRC [2, 4, 18, 19]. The reduction in risk was apparent across all age groups, and was unrelated to dose, indication, gender, CRC location or case-fatality status. We also found an effect of low-dose aspirin among patients who started therapy after having previously undergone colonoscopy/sigmoidoscopy. The protective effect was observed in Dukes Stages B to D CRC, occurring throughout treatment duration and starting within the first year of therapy, while a suggestion of a reduced risk of Dukes Stage A CRC was observed but only after 5 years’ therapy.

To our knowledge, our study is the first to investigate the effect of low-dose aspirin on risk of CRC by stage at diagnosis among real-world patients in clinical practice. A key strength of our study is the original observational study design, which attempted to minimize bias between aspirin users and non-users at start of follow-up that are difficult to control. By obtaining incident cases of CRC for our case–control analysis from two cohorts of patients – new users of low-dose aspirin and non-users of low-dose aspirin at start of follow-up – matched by factors including a proxy for general health status, we attempted to control for this possible selection bias. This would not have been achieved from a traditional nested case–control study design in the whole THIN source population, following up the single cohort to identify cases of CRC. By then analyzing low-dose aspirin in our case-control analysis in relation to the index date (i.e. current use, never use etc), we evaluated actual use of low-dose aspirin (assuming the patient took the medication) rather than use of low-dose aspirin defined at start of follow-up, which could have changed substantially over time. Other study strengths include the representativeness of the data source to the UK population, the long study period, and the inclusion of a broad range of patients, including those with gastrointestinal disorders. Lag-time and stratified analyses showed the associations to be robust and to occur across patient groups. All incident CRC cases were identified following thorough review of EMRs, previous validation of the CRC diagnosis of nearly 20% of our cases using record linkage to hospitalization data, and through PCP questionnaires for a smaller sample, resulting in high confirmation rates [13]. Any potential differential misclassification of CRC cases between low-dose aspirin users and non-users, for instance diagnostic bias arising from more frequent investigation and referral of aspirin users, would have underestimated the associations found. Although information on Dukes Stage was unavailable for 53% of cases, the distribution of Dukes Stage in cases with a recorded stage is largely consistent with national data [20, 21] supporting their representativeness to cases in the general population. Furthermore, 1- and 5-year survival rates for all cases are consistent with national rates [22]. Through sensitivity analysis restricted to CRC cases with known stage, we showed the associations to be robust. Misclassification of aspirin exposure owing to unrecorded OTC aspirin in THIN was likely to have been minimal, as shown in our previous validation study of low-dose aspirin recording in THIN [16] and other populations [23]. It is unlikely that our findings can be explained by healthy-user bias because we found the new users of low-dose aspirin cohort to be less healthy in terms of comorbidities and lifestyle factors than the matched (at start of follow-up) cohort. In fact, any residual lack of adjustment for the more severe co-morbidity profile of new low-dose aspirin users would have led to an underestimation of the protective effect seen. Furthermore, in another study using a similar design but where the non-user at start of follow-up cohort instead comprised new users of a ‘neutral’ drug – paracetamol – we report highly similar results [24]. Also, in this current study, we matched our two study cohorts by number of PCP visits as a proxy for general health/comorbidity. We controlled for several confounders in our analyses, and the level of missing data for smoking and body mass index was low with risk estimates virtually similar when these variables were ascertained before the start of follow-up (data not presented). Although data on certain CRC risk factors such as red meat intake and a positive family history and were not recorded; these are unlikely to be related to aspirin exposure and confound the associations observed. Residual confounding, however, cannot be ruled out. In addition, because the mean length of low-dose aspirin use was 6 years, we were unable to investigate the effect of low-dose aspirin when used in the longer-term.

Our findings support an effect of low-dose aspirin on at least two phases of the natural history of CRC. The early and large reduction in risk seen among patients presenting with Dukes Stage D CRC is consistent with previous clinical trial data [4]. Experimental evidence exists to suggest that platelets play an important role in metastasis [25, 26]. Pharmacological inhibition of thromboxane synthase in animal models has been shown to significantly inhibit tumour cell growth, invasion, metastasis and angiogenesis [27]. The short-term effect of aspirin on advanced stage CRC seen in our study supports the hypothesis that aspirin has an effect on the progression of established tumours – a clinically important finding because of the poor prognosis of patients presenting with advanced stage disease. Five-year survival for Dukes Stage D CRC cases in our study was 9%, in line with previous reports [20]. At least 5 years’ therapy with low-dose aspirin was necessary to observe a reduction in risk of Dukes A CRC, a latency period consistent with the average time for an early stage carcinoma to evolve from an adenomatous polyp [28]. This adds supports for an effect of aspirin early in the adenoma–carcinoma sequence [3]. Biologically plausible mechanisms that could mediate an effect of aspirin against CRC development have been postulated, also involving platelet inhibition [29]. Co-incubation of activated human platelets with CRC cells has been shown to upregulate cyclooxygenase-2 (COX-2) expression and induce epithelial-mesenchymal transition [30]. Induction of COX-2 expression in adjacent nucleated cells could thereby trigger a chain of downstream events affecting cell proliferation, apoptosis and angiogenesis. At low-doses (75–100 mg daily), aspirin permanently inactivates the enzyme cyclooxygenase 1 in platelets, leading to long-lasting suppression of thromboxane A2 production.

For Dukes Stages B to D CRC, we did not observe the latency period for an effect of low-dose aspirin as seen in post-hoc analyses of trial data [2, 18]. And interestingly, in a recent analysis of data from the Women’s Health Initiative [31] use of statins – medication also reported as having anti-cancer properties [32] – was found to be associated with a significant reduction in CRC mortality. There are several possible reasons why we were able to identify cases earlier during follow-up than previous aspirin trials. Firstly, our study was designed to investigate CRC as the clinical endpoint, and information on low-dose aspirin use throughout the whole follow-up was ascertained and analyzed. Secondly, our study did not screen-out patients with peptic ulcer disorders or other disease-related eligibility criteria, and some of these patients may have had preclinical CRC, already along the lengthy adenoma–carcinoma sequence at start of follow-up. Thirdly, our study population included real-world patients, which included those with multiple comorbidities; the average age of patients at start of follow-up was 64 years in our study compared with 61 in four previous low-dose aspirin trials [2]. Fourthly, follow-up began between 2000 and 2009, considerably later than the start of several trials that began in early/mid 1980s. Irrespective of the age and health of the study population, there have been temporal changes in the management of CRC, including the availability of better diagnostic techniques, the introduction of multidisciplinary teams in secondary care, the national bowel cancer screening programme, bowel cancer awareness initiatives/increased media attention, and accessibility to information.

In line with trial data [2], we found an aspirin dose of 75 mg/day to be effective in reducing CRC. No increased benefit was seen with up to four-fold higher aspirin doses, although our study was underpowered to reliably evaluate these exposures with around 90% of current low-dose aspirin use at 75 mg/day. Nevertheless, this is an important finding considering that doses of less than 100 mg are sufficient to reduce the risk of thrombotic cardiovascular events, and the dose-dependent increased risk of gastrointestinal bleeding [33]. The benefits of low-dose aspirin in reducing ischaemic vascular events [34] must be weighed against the risk of gastrointestinal bleeding, which increases with age and when aspirin is prescribed alongside other gastrotoxic agents [33]. Prevention strategies to minimize aspirin-associated gastrointestinal problems, such as use of proton pump inhibitors, have conferred an acceptable safety profile for low-dose aspirin use in the general population and thereby the potential for use in CRC prophylaxis. Recently, the U.S. Preventive Services Task Force issued a draft recommendation statement advocating long-term use of low-dose aspirin for primary prevention of CRC and CVD among adults aged 50 to 59 years who have a 10% or greater 10-year CVD risk and who are not at increased risk of bleeding [35]. For comparable adults aged 60 to 69 years, the Task Force recommends the decision to take low-dose aspirin for primary CRC and CVD prevention should be made on an individual basis. A quantification of the gastrointestinal safety profile of low-dose aspirin in our patient population is warranted to make a valid benefit/risk evaluation in CRC prevention. There is also a need for our results for CRC stage to be verified in other large, population-based, observational studies. The potential for low-dose aspirin to be used post-diagnosis as adjuvant treatment to prevent recurrence and prolong survival is another promising research question and is currently being assessed in the ongoing placebo-controlled ADD-Aspirin trial [36].

## Conclusions

Our results indicate that patients starting low-dose aspirin therapy have a reduced risk of Stages B–D CRC suggesting a role for low-dose aspirin in the progression of established CRC. When used in the long-term (5 years or more), low-dose aspirin may also substantially reduce the risk of Dukes A CRC.

## Additional files

Additional file 1: Supplementary Methods. (DOCX 19 kb)

Additional file 2: Table S1. Read codes for CRC. (DOCX 18 kb)

Additional file 3: Table S2. Clinical features of CRC cases. (DOCX 19 kb)

Additional file 4: Table S3. Average follow-up time from start date to CRC date by Dukes Stage. (DOCX 18 kb)

Additional file 5: Table S4. Characteristics of CRC cases with recorded stage and CRC cases with unknown stage. (DOCX 22 kb)

Additional file 6: Table S5. RRs (95% CI) for the risk of CRC by duration of low-dose aspirin use according to patient sub-groups. (DOCX 24 kb)

Additional file 7: Table S6. RRs (95% CI) for the risk of CRC by duration of low-dose aspirin use according to patient sub-groups. (DOCX 21 kb)

Additional file 8: Table S7. Frequency distribution of low-dose aspirin among CRC cases with recorded stage and controls, and RR (95% CI) for their association with risk of CRC. (DOCX 20 kb)

Additional file 9: Table S8. RRs (95% CI) for the risk of CRC associated with use of low-dose aspirin: 1-year lag time analysis. (DOCX 19 kb)

## Abbreviations

CI: Confidence interval
COX-2: Cyclooxygenase-2
CPRD: Clinical Practice Research Datalink
CVD: Cardiovascular disease
EMR: Electronic medical records
HES: Hospital Episode Statistics
OTC: Over-the-counter
PCP: Primary care practitioner
RCT: Randomized controlled trial
THIN: The Health Improvement Network
UK: United Kingdom
