# Supplementary material for: New use of low-dose aspirin and risk of colorectal cancer by stage at diagnosis: a nested case–control study in UK general practice
Source: BMC Cancer. 2017 Sep 7;17:637. doi: 10.1186/s12885-017-3594-9 (PMC5590216; doi:10.1186/s12885-017-3594-9)
Supplement: Supplementary file 1 — Supplementary Methods. (DOCX 19 kb) [file 12885_2017_3594_MOESM1_ESM.docx]

**Supplementary Methods**

Identification and descriptive analysis of the two study cohorts.

To identify the two study cohorts – a cohort of new users of low-dose aspirin at the start of follow-up (start date) and a cohort of 1:1 matched individuals still free of low-dose aspirin at the start date – individuals in the source population were followed from the date of meeting all eligibility criteria to the earliest of the following: first prescription for low-dose aspirin, diagnosis of cancer, age 85 years, death, or 31 December 2009. Patients censored as new users of low-dose aspirin were assigned to the low-dose aspirin cohort on that day (the start date). Descriptive analyses were undertaken to examine characteristics of the two study cohorts at the start of follow-up. All variables were measured any time before the start of follow-up (taking the most recent record before the start date) except for PCP visits, which were collected in the year before the start of follow-up.

Assessment of potential risk factors

Information on potential risk factors was extracted from the database, including demographics, body mass index, lifestyle factors and morbidities (any time before the index date, using the most recent record before the index date), PCP visits, referrals and hospitalizations (in the year before the index date), and medication use (any time before the index date). To ascertain the indication for low-dose aspirin, we used a computer algorithm that searched THIN for Read codes suggestive of cardiovascular disease (CVD; comprising myocardial infarction, unstable angina, peripheral artery disease and ischaemic heart disease) from any time before the first low-dose aspirin prescription and up to 30 days after. Patients with a relevant Read code within this time frame were classed as having received low-dose aspirin for secondary CVD prevention and all other patients were assumed to have received low-dose aspirin for primary CVD prevention. Information was also extracted from the database regarding previous bowel diagnostic imagining investigations (record of an adenoma, colonoscopy or sigmoidoscopy) or upper gastrointestinal disorders (dyspepsia, or complicated or uncomplicated peptic ulcer) any time before the start date, as well as the following medications: antihypertensives, statins, antidiabetics, non-steroidal anti-inflammatory drugs (NSAIDs), traditional NSAIDs, cyclooxygenase-2 selective inhibitors, oral steroids, warfarin, thienopyridines and proton pump inhibitors.
