# Supplementary material for: New use of low-dose aspirin and risk of colorectal cancer by stage at diagnosis: a nested case–control study in UK general practice
Source: BMC Cancer. 2017 Sep 7;17:637. doi: 10.1186/s12885-017-3594-9 (PMC5590216; doi:10.1186/s12885-017-3594-9)
Supplement: Supplementary file 2 — Read codes for CRC. (DOCX 18 kb) [file 12885_2017_3594_MOESM2_ESM.docx]

**Table S1.** Read codes for CRC

| **Read** | **Descriptor** |
| --- | --- |
| B13..00 | Malignant neoplasm of colon |
| B130.00 | Malignant neoplasm of hepatic flexure of colon |
| B131.00 | Malignant neoplasm of transverse colon |
| B132.00 | Malignant neoplasm of descending colon |
| B133.00 | Malignant neoplasm of sigmoid colon |
| B134.00 | Malignant neoplasm of caecum |
| B134.11 | Carcinoma of caecum |
| B136.00 | Malignant neoplasm of ascending colon |
| B137.00 | Malignant neoplasm of splenic flexure of colon |
| B138.00 | Malignant neoplasm, overlapping lesion of colon |
| B13y.00 | Malignant neoplasm of other specified sites of colon |
| B13z.00 | Malignant neoplasm of colon NOS |
| B13z.11 | Colonic cancer |
| B14..00 | Malignant neoplasm of rectum, rectosigmoid junction and anus |
| B140.00 | Malignant neoplasm of rectosigmoid junction |
| B141.00 | Malignant neoplasm of rectum |
| B141.11 | Carcinoma of rectum |
| B141.12 | Rectal carcinoma |
| B14y.00 | Malig neop other site rectum, rectosigmoid junction and anus |
| B14z.00 | Malignant neoplasm rectum, rectosigmoid junction and anus NOS |
| ZV10017 | [V]Personal history of malignant neoplasm of rectum |
| 8Hn4.00 | Fast track referral for suspected colorectal cancer |
| 9Np7.00 | Seen in fast track suspected colorectal cancer clinic |
