# Supplementary material for: New use of low-dose aspirin and risk of colorectal cancer by stage at diagnosis: a nested case–control study in UK general practice
Source: BMC Cancer. 2017 Sep 7;17:637. doi: 10.1186/s12885-017-3594-9 (PMC5590216; doi:10.1186/s12885-017-3594-9)
Supplement: Supplementary file 3 — Clinical features of CRC cases. (DOCX 19 kb) [file 12885_2017_3594_MOESM3_ESM.docx]

**Table S2.** Clinical features of CRC cases.

| **Characteristic** | **N=3033**  **n (%)** |
| --- | --- |
| **Type** | |
| Colon  Rectum^*^  More than one type | 1877 (61.9)  1111 (36.6)  45 (1.5) |
| **Site for colon cancer**^†^  Right  Left  Unknown | 894 (47.6)  607 (32.3)  376 (20.1) |
| **Symptoms recorded** | |
| Yes | 2214 (73.0) |
| No  Part of screening programme  **Symptoms**  Diarrhoea  Change in bowel habits  Anaemia  Constipation  Abdominal pain  Weight loss  Bleeding per rectum  Others  Mass  Obstruction | 712 (23.5)  107 (3.5)  2214 (73.0)  291 (13.1)  331 (15.0)  528 (23.8)  151 (6.8)  379 (17.1)  90 (4.1)  648 (29.3)  103 (4.7)  29 (1.3)  21 (0.9) |
| **Dukes Stage** | |
| Dukes A  Dukes B  Dukes C  Dukes D  Unknown | 178 (5.9)  377 (12.4)  370 (12.2)  496 (16.4)  1612 (53.2) |
| **Any diagnostic procedure** | 2036 (67.1) |
| **Type** |  |
| Colonoscopy | 1223 (60.1) |
| Sigmoidoscopy | 564 (27.7) |
| Barium enema | 228 (11.2) |
| Faecal occult blood test | 179 (8.8) |
| Abdominal ultrasound | 89 (4.4) |
| CAT Scan | 294 (14.4) |
| Other^‡^ | 53 (2.6) |

Percentages for CRC site, individual symptoms, and individual diagnostic procedures are among the total number of the cases with a record of the respective variable.

^*^Recto-sigmoid was considered as situated in the rectum.

^†^Right colon included from caecum and proximal colon to splenic flexure; left colon included distal to splenic flexure, including the splenic colon.

^‡^Includes endoscopy and rectum examination unspecified.

CAT, computed aided tomography; CRC, colorectal cancer.
