# Supplementary material for: New use of low-dose aspirin and risk of colorectal cancer by stage at diagnosis: a nested case–control study in UK general practice
Source: BMC Cancer. 2017 Sep 7;17:637. doi: 10.1186/s12885-017-3594-9 (PMC5590216; doi:10.1186/s12885-017-3594-9)
Supplement: Supplementary file 4 — Average follow-up time from start date to CRC date by Dukes Stage. (DOCX 18 kb) [file 12885_2017_3594_MOESM4_ESM.docx]

**Table S3.** Average follow-up time from start date to CRC date by Dukes Stage.

| **Dukes Stage** | **Mean follow-up time (years)** | **Median follow-up time  (years [interquartile range])** |
| --- | --- | --- |
| A | 3.16 | 2.85 (1.02 – 4.77) |
| B | 3.10 | 2.54 (0.98 – 4.60) |
| C | 3.14 | 2.69 (1.10 – 6.91) |
| D | 3.16 | 2.73 (1.01 – 6.72) |
| Unknown | 3.42 | 2.87 (1.21 – 5.11) |
