# Supplementary material for: New use of low-dose aspirin and risk of colorectal cancer by stage at diagnosis: a nested case–control study in UK general practice
Source: BMC Cancer. 2017 Sep 7;17:637. doi: 10.1186/s12885-017-3594-9 (PMC5590216; doi:10.1186/s12885-017-3594-9)
Supplement: Supplementary file 5 — Characteristics of CRC cases with recorded stage and CRC cases with unknown stage. (DOCX 22 kb) [file 12885_2017_3594_MOESM5_ESM.docx]

**Table S4.** Characteristics of CRC cases by stage at diagnosis.

|  | **Dukes A**  **N=178**  **n (%)** | **Dukes B**  **N=377**  **n (%)** | **Dukes C**  **N=370**  **n (%)** | **Dukes D**  **N=496**  **n (%)** | **Unknown stage**  **N=1612**  **n (%)** |
| --- | --- | --- | --- | --- | --- |
| **Gender** |  |  |  |  |  |
| Male | 104 (58.4) | 226 (59.9) | 224 (60.5) | 320 (64.5) | 932 (57.8) |
| Female | 74 (41.6) | 151 (50.1) | 146 (39.5) | 176 (35.5) | 680 (42.2) |
| **Age group (years)** |  |  |  |  |  |
| 40–49 | 2 (1.1) | 1 (0.3) | 2 (0.5) | 3 (0.6) | 11 (0.7) |
| 50–59 | 13 (7.3) | 22 (5.8) | 40 (10.8) | 47 (9.5) | 118 (7.3) |
| 60–69 | 62 (34.8) | 108 (28.6) | 126 (34.1) | 144 (29.0) | 435 (27.0) |
| 70–79 | 81 (45.5) | 189 (50.1) | 138 (37.3) | 205 (41.3) | 660 (40.9) |
| ≥80 | 20 (11.2) | 57 (15.1) | 64 (17.3) | 97 (19.6) | 388 (24.1) |
| **Year of index date** |  |  |  |  |  |
| 2000–2003 | 26 (14.6) | 68 (18.0) | 51 (13.8) | 59 (11.9) | 224 (13.9) |
| 2004–2006 | 56 (31.5) | 106 (28.1) | 120 (32.4) | 150 (30.2) | 386 (23.9) |
| 2007–2009 | 96 (53.9) | 203 (53.8) | 199 (53.8) | 287 (57.9) | 1002 (62.2) |
| **Symptoms/signs in the year prior index date** |  |  |  |  |  |
| Polyp/adenoma | 31 (17.4) | 14 (3.7) | 24 (6.5) | 29 (5.8) | 106 (6.6) |
| Rectal bleeding | 51 (28.7) | 52 (13.8) | 58 (15.7) | 51 (10.3) | 262 (16.3) |
| Diarrhoea | 15 (8.4) | 45 (11.9) | 49 (13.2) | 71 (14.3) | 183 (11.4) |
| Change in bowel habit | 20 (11.2) | 54 (14.3) | 46 (12.4) | 51 (10.3) | 172 (10.7) |
| Weight loss | 4 (2.2) | 11 (2.9) | 7 (1.9) | 21 (4.2) | 54 (3.3) |
| Constipation | 3 (1.7) | 24 (6.4) | 21 (4.2) | 27 (5.4) | 76 (4.7) |
| Abdominal pain | 6 (3.4) | 48 (12.7) | 70 (14.1) | 86 (17.3) | 169 (10.5) |
| Anaemia | 17 (9.6) | 72 (19.1) | 47 (12.7) | 61 (12.3) | 218 (13.5) |
| Malaena | 4 (2.2) | 3 (0.8) | 0 (0) | 3 (0.6) | 7 (0.4) |
| **Diagnostic tests in the year prior index date** |  |  |  |  |  |
| Colonoscopy | 61 (34.3) | 82 (21.8) | 74 (20.0) | 101 (20.4) | 359 (22.3) |
| Sigmoidoscopy | 45 (25.3) | 54 (14.3) | 49 (13.2) | 48 (9.7) | 182 (11.3) |
| Barium enema | 8 (4.5) | 24 (6.4) | 27 (7.3) | 17 (3.4) | 69 (4.3) |
| FOB test | 19 (10.7) | 35 (9.3) | 39 (10.5) | 32 (6.5) | 164 (10.2) |

FOB, faecal occult blood test.
