# Supplementary material for: New use of low-dose aspirin and risk of colorectal cancer by stage at diagnosis: a nested case–control study in UK general practice
Source: BMC Cancer. 2017 Sep 7;17:637. doi: 10.1186/s12885-017-3594-9 (PMC5590216; doi:10.1186/s12885-017-3594-9)
Supplement: Supplementary file 6 — RRs (95% CI) for the risk of CRC by duration of low-dose aspirin use according to patient sub-groups. (DOCX 24 kb) [file 12885_2017_3594_MOESM6_ESM.docx]

**Table S5.** Characteristics of CRC cases with recorded stage and CRC cases with unknown stage.

|  | **Recorded stage**  **N=1421**  **n (%)** | **Unknown stage**  **N=1612** |
| --- | --- | --- |
| **Type of CRC** |  |  |
| Colon | 932 (65.6) | 945 (58.6) |
| Rectum^*^ | 487 (34.3) | 624 (38.7) |
| More than one type | 2 (1.4) | 43 (2.7) |
| **Site for colon cancer**^†^ | 932 | 945 |
| Right | 476 (51.1) | 418 (44.2) |
| Left | 314 (33.7 | 293 (31.0) |
| Unknown | 142 (15.2) | 234 (24.8) |
| **Symptoms/signs recorded in the year prior index date** |  |  |
| Yes | 1089 (76.6) | 1125 (69.8) |
| No | 483 (20.5) | 420 (26.0) |
| Part of screening programme | 40 (2.8) | 67 (4.2) |
| **Symptoms/signs in the year prior index date** |  |  |
| Polyp/adenoma | 98 (6.9) | 106 (6.6) |
| Rectal bleeding | 212 (14.9) | 262 (16.3) |
| Diarrhoea | 180 (12.7) | 183 (11.4) |
| Change in bowel habit | 171 (12.0) | 172 (10.7) |
| Weight loss | 43 (3.0) | 54 (3.4) |
| Constipation | 76 (5.3) | 75 (4.7) |
| Abdominal pain | 169  (11.9) | 210 (13.0) |
| Anaemia | 197 (13.9) | 258 (13.5) |
| Malaena | 25 (1.8) | 20 (1.2) |
| **Diagnostic tests in the year prior index date** |  |  |
| Colonoscopy | 318 (22.3) | 359 (22.3) |
| Sigmoidoscopy | 196 (13.8) | 182 (11.3) |
| Barium enema | 76 (5.4) | 69 (4.3) |
| FOB test | 125 (8.8) | 164 (10.2) |
| **Comorbidities^*^** |  |  |
| Hypertension | 797 (56.1) | 943 (58.5) |
| Diabetes | 263 (18.5) | 335 (20.8) |
| Hypercholesterolaemia | 220 (15.5) | 264 (16.4) |
| IBD | 333 (20.7) | 257 (18.1) |
| IBS | 97 (6.0) | 79 (5.6) |
| UGI disorders | 253 (17.8) | 268 (16.6) |
| **Smoking^†^** |  |  |
| Non-smoker | 581 (40.9) | 650 (40.3) |
| Current smoker | 175 (12.3) | 201 (12.5) |
| Former smoker | 634 (44.6) | 728 (45.2) |
| Unknown | 31 (2.2) | 33 (2.0) |
| **Alcohol consumption (u/w)^†^** |  |  |
| None | 220 (15.5) | 263 (16.3) |
| 1–9 | 672 (47.3) | 729 (45.2) |
| 10–20 | 232 (16.3) | 248 (15.4) |
| ≥21 | 121 (8.5%) | 153 (9.5%) |
| 21–41 | 97 (6.8) | 119 (7.4) |
| ≥42 | 24 (1.7) | 34 (2.1) |
| Unknown | 176 (12.4) | 219 (13.6) |
| **BMI^†^** |  |  |
| <20 | 47 (3.3) | 64 (4.0) |
| 20–24 | 371 (26.1) | 403 (25.0) |
| 25–29 | 562 (39.5) | 582 (36.1) |
| ≥30 | 316 (22.2) | 398 (24.7) |
| Unknown |  |  |
| **PCP visits**^‡^ |  |  |
| 0–1 | 36 (2.5) | 56 (3.5) |
| 2–4 | 118 (8.3) | 143 (8.9) |
| 5–9 | 337 (23.7) | 362 (22.5) |
| 10–19 | 548 (38.6) | 616 (38.2) |
| ≥20 | 382 (26.9) | 435 (27.0) |
| **Referrals**^‡^ |  |  |
| 0–1 | 537 (37.8) | 645 (40.0) |
| 2–4 | 431 (30.3) | 486 (30.1) |
| 5–9 | 297 (20.9) | 315 (19.5) |
| ≥10 | 156 (11.0) | 166 (10.3) |
| **Hospitalizations**^‡^ |  |  |
| None | 1137 (80.0) | 1287 (79.8) |
| 1 | 181 (12.7) | 194 (12.0) |
| 2 | 67 (4.7) | 81 (5.0) |
| ≥3 | 36 (2.5) | 50 (3.1) |

BMI, body mass index; COPD, chronic obstructive pulmonary disorder; FOB, faecal occult blood; GI, gastrointestinal; IBD, inflammatory bowel disease; IBS, irritable bowel syndrome; PCP, primary care practitioner; UGI, upper gastrointestinal disorders; u/w, units per week.

**^*^**Any time before the index date except for GI disorders, which were ascertained anytime up to and including the start date.

^†^Any time before the index date taking the value recorded nearest to the index date.

^‡^In the year before the index date.
