# Supplementary material for: New use of low-dose aspirin and risk of colorectal cancer by stage at diagnosis: a nested case–control study in UK general practice
Source: BMC Cancer. 2017 Sep 7;17:637. doi: 10.1186/s12885-017-3594-9 (PMC5590216; doi:10.1186/s12885-017-3594-9)
Supplement: Supplementary file 7 — RRs (95% CI) for the risk of CRC by duration of low-dose aspirin use according to patient sub-groups. (DOCX 21 kb) [file 12885_2017_3594_MOESM7_ESM.docx]

**Table S6.** RRs (95 % CI) for the risk of CRC by duration of low-dose aspirin use according to patient sub-groups.

| **Duration of  low-dose aspirin** | **Controls**  **N=10,000**  **n (%)** | | **Cases**  **N=3033**  **n (%)** | | **RR (95% CI)^*^** | **RR (95% CI)**^†^ |
| --- | --- | --- | --- | --- | --- | --- |
| **Primary CVD prevention** | | | | | | |
| <1 year | 700 (7.0) | | 221 (7.3) | | 0.76 (0.64 – 0.90) | 0.75 (0.64 – 0.89) |
| 1–5 years | 1518 (15.2) | | 433 (14.3) | | 0.70 (0.62 – 0.80) | 0.70 (0.61 – 0.79) |
| ≥5 years | 342 (3.4) | | 92 (3.0) | | 0.66 (0.52 – 0.84) | 0.65 (0.51 – 0.84) |
| **Secondary CVD prevention** | | | | | | |
| <1 year | 516 (5.2) | | 144 (4.7) | | 0.65 (0.53 – 0.79) | 0.65 (0.53 – 0.79) |
| 1–5 years | 1125 (11.2) | | 282 (9.3) | | 0.60 (0.51 – 0.69) | 0.59 (0.51 – 0.69) |
| ≥5 years | 361 (3.6) | | 83 (2.7) | | 0.56 (0.43 – 0.72) | 0.56 (0.43 – 0.72) |
| **Dukes Stage A** |  | |  | |  |  |
| <1 year | 1216 (12.2) | | 33 (18.5) | | 1.28 (0.82 – 2.00) | 1.28 (0.44 – 1.39) |
| 1–5 years | 2643 (26.4) | | 46 (25.8) | | 0.87 (0.58 – 1.30) | 0.86 (0.57 – 1.28) |
| ≥5 years | 703 (7.0) | | 7 (3.9) | | 0.55 (0.24 – 1.23) | 0.53 (0.24 – 1.19) |
| **Dukes Stage B** |  | |  | |  |  |
| <1 year | 1216 (12.2) | | 48 (12.7) | | 0.63 (0.45 – 0.88) | 0.62 (0.44 – 0.87) |
| 1–5 years | 2643 (26.4) | | 81 (21.5) | | 0.53 (0.40 – 0.70) | 0.52 (0.39 – 0.68) |
| ≥5 years | 703 (7.0) | | 17 (4.5) | | 0.44 (0.26 – 0.73) | 0.42 (0.25 – 0.72) |
| **Dukes Stage C** |  |  |  |  |  |  |
| <1 year | 1216 (12.2) | | 37 (10.0) | | 0.60 (0.41 – 0.88) | 0.60 (0.41 – 0.87) |
| 1–5 years | 2643 (26.4) | | 100 (27.1) | | 0.80 (0.61 – 1.05) | 0.79 (0.60 – 1.04) |
| ≥5 years | 703 (7.0) | | 18 (4.9) | | 0.62 (0.37 – 1.04) | 0.61 (0.36 – 1.02) |
| **Dukes Stage D** |  |  |  |  |  |  |
| <1 year | 1216 (12.2) | | 57 (11.5) | | 0.64 (0.47 – 0.87) | 0.64 (0.47 – 0.87) |
| 1–5 years | 2643 (26.4) | | 112 (22.6) | | 0.59 (0.46 – 0.75) | 0.59 (0.46 – 0.75) |
| ≥5 years | 703 (7.0) | | 27 (5.5) | | 0.55 (0.36 – 0.83) | 0.55 (0.36 – 0.85) |
| **0–9 PCP visits in the year prior index date** | | | | | | |
| <1 year | 381 (9.1) | | 93 (8.8) | | 0.74 (0.58 – 0.95) | 0.73 (0.57 – 0.94) |
| 1–5 years | 852 (20.3) | | 152 (14.4) | | 0.59 (0.49–0.72) | 0.59 (0.48 – 0.71) |
| **≥**5 years | 195 (4.6) | | 36 (3.4) | | 0.68 (0.46 – 0.98) | 0.66 (0.45 – 0.97) |
| **≥ 10 PCP visits in the year prior index date** | | | | | | |
| <1 year | 835 (14.4) | | 272 (13.7) | | 0.80 (0.67 – 0.94) | 0.80 (0.67 – 0.94) |
| 1–5 years | 1791 (30.9) | | 563 (28.4) | | 0.75 (0.65 – 0.86) | 0.74 (0.64 – 0.85) |
| **≥**5 years | 508 (8.8) | | 139 (7.0) | | 0.63 (0.51 – 0.79) | 0.63 (0.51 – 0.79) |

**All estimates are among current users of low-dose aspirin (reference group = non-use) unless otherwise specified**.

^*^Adjusted by the matching factors and number of PCP visits.

^†^Adjusted by the matching factors, number of PCP visits, smoking (any time before index date), insulin, NSAIDs, BMI (any time before index date), oral steroids and low-dose aspirin.

BMI, body mass index; CI, confidence interval; CVD, cardiovascular disease; NSAIDS, non-steroidal anti-inflammatory drugs; PCP, primary care practitioner; RR, rate ratio.
