# Supplementary material for: New use of low-dose aspirin and risk of colorectal cancer by stage at diagnosis: a nested case–control study in UK general practice
Source: BMC Cancer. 2017 Sep 7;17:637. doi: 10.1186/s12885-017-3594-9 (PMC5590216; doi:10.1186/s12885-017-3594-9)
Supplement: Supplementary file 8 — Frequency distribution of low-dose aspirin among CRC cases with recorded stage and controls, and RR (95% CI) for their association with risk of CRC. (DOCX 20 kb) [file 12885_2017_3594_MOESM8_ESM.docx]

**Table S7**. Frequency distribution of low-dose aspirin among CRC cases with recorded stage and controls, and RR (95% CI) for their association with risk of CRC.

| **Low-dose aspirin use** | **Controls**  **N=10,000**  **n (%)** | | **Cases with recorded stage**  **N=1421**  **n (%)** | | **RR (95% CI)*** | **RR (95% CI)†** |
| --- | --- | --- | --- | --- | --- | --- |
| Recency |  |  |  |  |  |  |
| Non-use | 3557 (35.6) | | 592 (41.7) | | 1.00 (–) | 1.00 (–) |
| Current use | 4562 (45.6) | | 584 (41.1) | | 0.78 (0.69 – 0.88) | 0.64 (0.56 – 0.73) |
| Recent/past use‡ | 475 (4.8) | | 245 (17.3) | | 0.82 (0.69 – 0.96) | 0.68 (0.58 – 0.81) |
| Daily dose§ | | | | | | |
| 75 mg | 4128 (41.3) | | 540 (38.0) | | 0.80 (0.70 – 0.91) | 0.66 (0.58 – 0.75) |
| 150 mg | 402 (4.0) | | 42 (3.0) | | 0.61 (0.44 – 0.85) | 0.49 (0.35 – 0.68) |
| 300 mg | 32 (0.3) | | 2 (0.1) | | 0.38 (0.09 – 1.60) | 0.32 (0.07 – 1.33) |
| Formulation | | | | | | |
| Plain | 3,716 (37.2) | | 469 (33.0) | | 0.77 (0.67 – 0.87) | 0.63 (0.55 – 0.72) |
| Enteric coated | 846 (8.5) | | 115 (8.1) | | 0.83 (0.67 – 1.03) | 0.68 (0.55 – 0.85) |
| Duration of use | | |  | |  |  |
| <1 year | 1,430 (14.3) | | 200 (14.1) | | 0.83 (0.70 – 0.98) | 0.67 (0.56 – 0.81) |
| 1–5 years | 2,370 (23.7) | | 304 (21.4) | | 0.77 (0.67 – 0.90) | 0.64 (0.55 – 0.75) |
| ≥5 years | 762 (7.6) | | 80 (5.6) | | 0.67 (0.52 – 0.87) | 0.57 (0.44 – 0.73) |

All estimates are among current users of low-dose aspirin (reference group = non-use) unless otherwise specified.

*Adjusted by the matching variables (age, sex and year of index date).

†Adjusted by the matching variables (age, sex and year of index date) and number of PCP visits, smoking (any time before index date), insulin, NSAIDs, BMI (any time before index date) and oral steroids.

‡For patients with a duration of use of at least 1 year (25% of all recent/past users)

§Refers to the estimated average quantity dose.

BMI, body mass index; CI, confidence interval; NSAIDS, non-steroidal anti-inflammatory drugs; PCP, primary care practitioner; RR, rate ratio.
