# Supplementary material for: New use of low-dose aspirin and risk of colorectal cancer by stage at diagnosis: a nested case–control study in UK general practice
Source: BMC Cancer. 2017 Sep 7;17:637. doi: 10.1186/s12885-017-3594-9 (PMC5590216; doi:10.1186/s12885-017-3594-9)
Supplement: Supplementary file 9 — RRs (95% CI) for the risk of CRC associated with use of low-dose aspirin: 1-year lag time analysis. (DOCX 19 kb) [file 12885_2017_3594_MOESM9_ESM.docx]

**Table S8.** RRs (95 % CI) for the risk of CRC associated with use of low-dose aspirin: 1-year lag time analysis.

| **Low-dose aspirin use** | **Controls**  **N=8150**  **n (%)** | | **Cases**  **N=2339**  **n (%)** | | **RR (95% CI)^*^** | **RR (95% CI)^†^** |
| --- | --- | --- | --- | --- | --- | --- |
| **1-year backdated index date** | | | | | | |
| **Recency** |  |  |  |  |  |  |
| Non-use | 2812 (34.5) | | 874 (37.4) | | 1.00 (–) | 1.00 (–) |
| Current use | 3916 (48.0) | | 1086 (46.4) | | 0.85 (0.76 – 0.95) | 0.82 (0.74 – 0.92) |
| **Dose**^‡^ | | | | | | |
| 75 mg | 3660 (44.9) | | 1009 (43.1) | | 0.84 (0.76 – 0.94) | 0.82 (0.73 – 0.92) |
| 150 mg | 223 (2.7) | | 67 (2.9) | | 0.93 (0.70 – 1.23) | 0.89 (0.67 – 1.19) |
| 300 mg | 33 (0.4) | | 10 (0.4) | | 0.94 (0.46 – 1.93) | 0.92 (0.45 – 1.87) |
| **Duration** | | | | | | |
| <1 year | 1,338 (16.4) | | 375 (16.0) | | 0.87 (0.75 – 1.00) | 0.85 (0.74 – 0.98) |
| 1–5 years | 2,039 (25.0) | | 574 (24.5) | | 0.86 (0.76 – 0.98) | 0.84 (0.74 – 0.95) |
| ≥5 years | 539 (6.6) | | 137 (5.9) | | 0.75 (0.61 – 0.93) | 0.72 (0.58 – 0.89) |

**All estimates are among current users of low-dose aspirin (reference group = non-use) unless otherwise specified**.

Note: Cases and controls for whom the backdated date fell before their respective start date were excluded from the analysis, and information on low-dose aspirin and other variables was measured up to 1 year before the original index date in eligible case and controls.

^*^Adjusted by the matching factors and number of PCP visits.

**^†^**Adjusted by the matching factors, number of PCP visits, smoking (any time before index date), insulin, NSAIDs, BMI (any time before index date), oral steroids, and low-dose aspirin.

^‡^Dose refers to the dose of the last prescription before the index date.

BMI, body mass index; CI, confidence interval; NSAIDS, non-steroidal anti-inflammatory drugs; PCP, primary care practitioner; RR, rate ratio.
